# Primary scleral-fixated posterior chamber intraocular lenses in patients with congenital lens subluxation

**DOI:** 10.1186/s12886-021-02182-0

**Published:** 2021-11-29

**Authors:** Anjian Wang, Qi Fan, Yongxiang Jiang, Yi Lu

**Affiliations:** 1 Department of Ophthalmology, Eye & ENT Hospital of Fudan University, 83 Fenyang Road, Shanghai, 200031 China; 2 NHC Key Laboratory of Myopia (Fudan University), Key Laboratory of Myopia, Chinese Academy of Medical Sciences, Shanghai, 200031 China; 3 Shanghai Key Laboratory of Visual Impairment and Restoration, Shanghai, 200031 China

**Keywords:** Congenital lens subluxation, Long-term follow-up, Postoperative eye trauma, Scleral-fixated posterior chamber intraocular lens

## Abstract

**Objective:**

We investigated the long-term visual outcomes and ocular complications of primary scleral-fixated posterior chamber intraocular lenses (SF-PC-IOLs) in patients with congenital lens subluxation.

**Methods:**

We enrolled 53 patients (77 eyes) with congenital lens subluxation caused by ectopia lentis, Marfan syndrome, and Weill–Marchesani syndrome who underwent primary implantation of a SF-PC-IOL. All patients underwent a complete ophthalmic examination include visual acuity (VA), intraocular pressure (IOP), intraocular lenses (IOL) position, intraoperative complications and postoperative complications. Cox regression analysis and survival analysis were used to evaluate the risk factors for postoperative complications.

**Results:**

Seventy seven eyes from 53 patients were included. Mean age at surgery was 23 ± 20 years (5 to 67 years), with a mean follow-up of 39 ± 27 months (12 to 130 months). The best-corrected VA improved from 0.84 ± 0.55 to 0.26 ± 0.43 logarithms of the minimum angle of resolution (*p* < 0.001). Best-corrected VA improved postoperatively in 73 eyes (94%). The main causes of reduced vision after surgery were retinal pathologies and amblyopia. Complications included transient intraocular haemorrhage (2 eyes, 2.6%), early vitreous incarceration (2 eyes, 2.6%), retinal detachment (6 eyes, 7.8%) and IOL dislocation (3 eyes, 3.9%). Cox regression showed that postoperative eye trauma was a risk factor for long-term postoperative complications.

**Conclusion:**

SF-PC-IOLs provide good visual outcomes in patients with congenital lens subluxation. The SF-PC-IOLs showed good stability, except in patients suffering from postsurgical eye trauma.

## Introduction

Congenital lens subluxation is a relatively rare disease that may present in isolation (e.g. sporadic or familial ectopia lentis, ectopia lentis et pupillae, and aniridia) or in combination with a systemic disease (e.g. Marfan syndrome, homocystinuria, Weill–Marchesani syndrome, Ehlers–Danlos syndrome, sulphite oxidase deficiency, and hyperlysinaemia) [1]. Early onset lens dislocation always results is uncorrected intraocular astigmatism and ametropia, which affect visual function and cause amblyopia in children. The treatment of congenital lens subluxation is particularly complex in young patients and in patients with poorly developed zonules [2].

Patients with mild lens subluxation or lens edge dislocation out of the visual axis can often be treated via optical correction (glasses or contact lenses) [3]. However, intolerance to contact lenses and anisometropia associated with glassesmean these approaches may be unviable in some patients. Furthermore, patients with very severe or progressive lens displacement and patients with complications may require surgical intervention. For severe lens subluxation, the surgical procedure is complicated and carries a high risk of complications. Furthermore, the best surgical approach is still debated. .

There are currently several surgical methods to remove the subluxated lens and rehabilitate the eye’s visual function. After lensectomy or extraction, IOLs can be implanted into the anterior chamber (AC-IOL), fixed on the iris, or implanted into the posterior chamber (PC-IOL) with sutured or non-sutured glue scleral-fixated [4]. Sutured Scleral-fixated PC-IOLs (SF-PC-IOL) have been used to treat lens subluxation for many years. Multiple case-series reports have also shown favourable short- to mid-term outcomes of SF-PC-IOL implantation [5–7]. Recently, Cionni CTR (Capsular Tension Ring) + PC-IOL implantation has attracted the attention because of its advantages of reducing the posterior eye segmental perturbation by keeping the posterior capsule, especially in children and young adults. However, complex surgical techniques and mCTR (modified CTR = Cionni CTR) as a special implanting device makes a difficult access to the hospitals in grassroots areas and areas where modified CTR are not available. Under these circumstances, primary SF-PC-IOL implantation remains a major surgical intervention in the correction of aphakic eyes without sufficient capsular support.

Therefore, the purpose of this study was to retrospectively evaluate the long-term visual outcomes and complications of patients with congenital lens subluxation who underwent primary SF-PC-IOL implantation.

## Methods

The Institutional Review Board of the Eye and ENT Hospital of Fudan University approved the protocol of this retrospective analysis study (the ethical approval reference number: No. 2013021). All procedures adhered to the tenets of the Declaration of Helsinki. All of the patients or guardians provided written informed consent for the surgical procedures and for the use of their medical records for research purposes.

### Subjects

We performed a retrospective review of the medical records of 77 eyes in 53 patients (31 males and 22 females) who underwent SF-PC-IOL implantation between January 2005 and June 2014 at the Eye & ENT Hospital of Fudan University, Shanghai, China. All eyes included in this study lacked the support of a continuous lens capsule in > 4 clock-face hours. The SF-PC-IOLs were implanted at the time of surgery for lensectomy and anterior vitrectomy to treat lens subluxation. After surgery, patients aged < 7 years old who had unilateral lens subluxation underwent treatment of amblyopia by patching the healthy eyes.

Either eyes of patients were excluded if RD or other complications occurred before the primary operation. Eyes were also excluded if they had a history of ocular surgery or received post- operative follow-up for fewer than 12 months.

### Clinical measurements

The clinical assessments included best-corrected visual acuity (BCVA), refraction, sensorimotor examination of eye alignment/eye movement, measurement of intraocular pressure (IOP), slit-lamp examination, fundoscopy, keratometry before and after surgery, assessment of the IOL position after surgery.

After surgery, anterior segment optical coherence tomography (AS-OCT; (CASIA2 anterior segment OCT; Tomey, Nagoya, Japan) was performed to analyse the IOL’s decentration and tilt using the anterior segment single-scan mode. Cross-sectional images of the IOL were obtained. IOL dislocation was defined as decentration of > 1 mm and IOL tilt was defined as a tilt angle of > 5° [8, 9]. The distance between the optical axis of the eye and the centre of the horizontal axis of the lens was calculated using Matlab (Mathworks, Natick, MA, USA), and the level of decentration was determined using this distance. The angle between the optical axis of the eye and the vertical axis of the lens was also calculated using Matlab, and was used to determine the tilt angle.

### Surgical technique

Before surgery, informed consent was obtained from the patients or their guardians. All surgeries were done by one surgeon (LY) and SF-PC-IOLs were implanted using the following technique.

Conjunctival peritomy were performed near two sides of corneoscleral limbus first. A transscleral suture was placed through the bed of two scleral flaps (4:00 and 10:00 positions or 2:00 and 8:00 positions) using the classic *ab externo* technique with a double-armed 10–0 polypropylene suture (Prolene; Ethicon Inc., Somerville, NJ). Then the loop of the suture was externalised through a 2.6-mm temporal clear corneal incision at 12:00 position with a hook and cut in the middle. The suture was either tied to the two haptic of IOL, and the haptics were drawn into the ciliary sulcus. The external knot was covered by the scleral flap. The scleral flap was sutured flat with a 10–0 nylon suture before conjunctival closure with a 8–0 nylon suture.

Following four kinds of PC-IOLs were used for scleral fixation in this cohort of patients: 1. HumanOptics model MCX11ASP (HumanOptics AG, Erlangen, Germany), a single-piece hydrophilic acrylic plate lens with 7.0 to 5.5 mm diameter optic and two modified frame haptics. 2. Alcon model CZ70BD (Alcon, Fort Worth, TX, USA), a single-piece polymethylmethacrylate lens with 7.0 mm diameter optic and two modified C-style haptics desighed with eyelet on it. 3. Rayner model 920H (Rayner, UK), a single-piece hydrophilic acrylic lens with 6.25 mm overall length of optic and two closed loop designed C-style haptics. 4. Abbott Medical Optics model AR40e (Abbott Medical Optics, Santa Ana, CA, USA), a three-piece hydrophobic acrylic lens with 6 mm diameter optic and two 60% Blue core PPMA modified C-style haptics.

### Statistical analysis

All analyses were performed using SPSS version 20.0 (SPSS, Chicago, Illinois, USA). Follow-up times were counted according to the unit of eyes due to different operation time of bilateral primary SF-PC-IOL implantation. Patients’ gender and etiology are expressed as the number and percent of patients. All clinical measurements (included BCVA, IOP, postoperative complications) are expressed as the number and percent of eyes. Continuous variables are expressed as the mean ± standard deviation (SD). Paired *t* tests were used to compare the preoperative and postoperative variables. Independent samples t test, one-way ANOVA analysis and Chi-Square tests were used to compare in subgroups. Cox regression analysis and survival analysis were used to identify risk factors for postoperative complications. Values of *p* ≤ 0.05 were considered statistically significant.

## Results

### Baseline characteristics

**Fig. 1:**
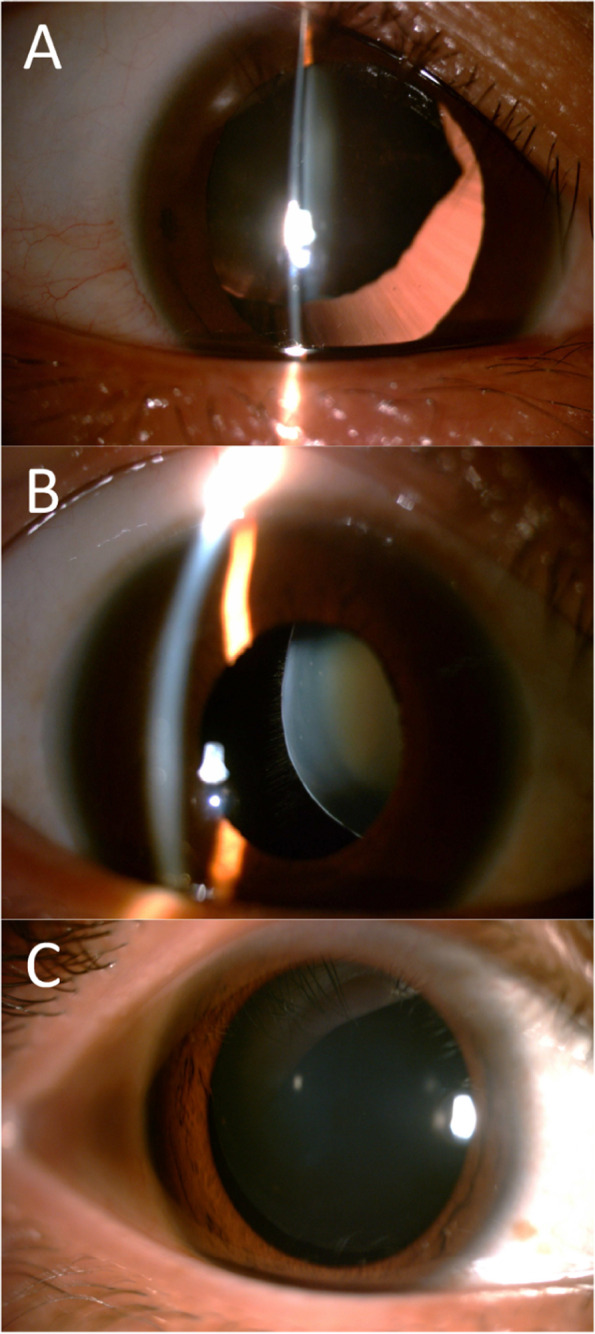
Slit-lamp photographs for 3 candidate in this study. **A** A subluxated lens with simple ectopia lentis; **B** A subluxated lens with Marfan’s syndrome; **C** A spherophakic lens with ectopia lentis in Weill-Marchesani syndrom. Written permission were obtained from patients for publication of slit-lamp photographs

The medical records for a total of 77 eyes in 53 patients (31 males and 22 females) were retrieved and analysed in this study. The mean follow-up time (FU) was 39 ± 27 months (range 12 to 130 months), > 5 years in 18 eyes (23%) and > 10 years in 4 eyes (5%). Relevant ophthalmological history included simple ectopia lentis 62%, lens subluxation due to Marfan’s syndrome 32%, and Weill-Marchesani syndrome 6% of all cases. Table 1 summarises the patient characteristics. The representative images of the slit lamp are presented in Fig. 1.

**Table 1:** Summary of patient demographics and follow-up time

Parameter	Value
Age, year	
Mean ± SD	23 ± 20
Range	5–67
Gender, n(%)	
Male	31(58%)
Female	22(42%)
Etiology (numbers of patients), (percentage)	
Simple Ectopia Lentis	33(62%)
Marfan’s Syndrome	17(32%)
Weill-Marchesani Syndrom	3 (6%)
Follow up duration, months	
Mean ± SD	39 ± 27
Range	12–130

*SD* Standard deviation

### Clinical outcomes

**Fig. 2:**
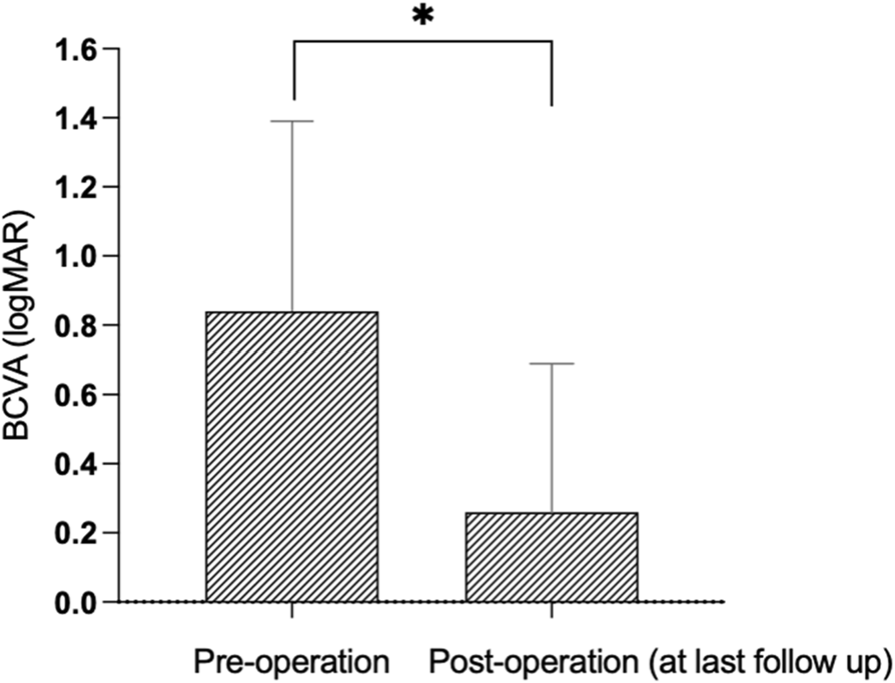
Changes of visual acuity before and after operation. The mean BCVA significantly improved after surgery at last follow-up in patients (*P* < 0.001). * *P* < 0.05 between the two groups

**Fig. 3:**
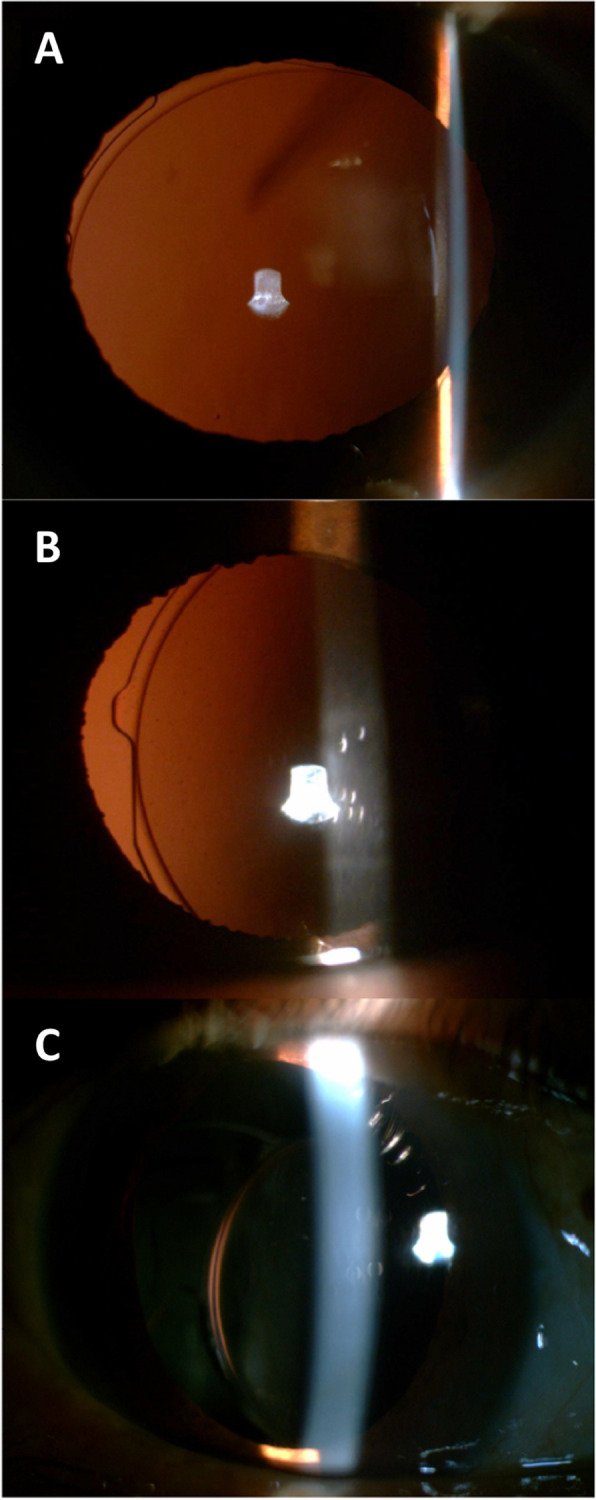
Photographs from slit-lamp examinations for IOL position. **A** At 9 years postoperatively, with no dislocation; **B** At 10 years postoperatively, with slight dislocation; **C** At 5 years postoperatively, with IOL dislocation required re-operation. IOL = intraocular lens. Written permission were obtained from those 3 patients for publication of slit-lamp photographs for IOL position

The mean ± SD BCVA (in logarithms of the minimum angle of resolution) improved significantly from 0.84 ± 0.55 (range 0.05 to 3.20) before surgery to 0.26 ± 0.43 (range − 0.20 to 3.00) at the last visit (*p* < 0.001) (Fig. 2). The BCVA improved after surgery in 73 eyes (94%) compared with the BCVA before surgery. For patients without an improvement in BCVA after surgery, this was due to retinal pathologies in most cases. We also compared the mean change in BCVA after surgery among patients divided into subgroups based on their initial ophthalmological history (i.e. simple ectopia lentis, Marfan syndrome, and Weill–Marchesani syndrome), while taking the preoperative BCVA into consideration, but found no difference among the three groups of patients (One Way ANOVA, *p* = 0.5). The BCVA before surgery was not significantly different between the pediatric (≤ 18 years old) group and adult (> 18 years old) group (*p* = 0.526). The BCVA after surgery was marginally significant different (*p* = 0.055), which was 0.17 ± 0.20 in pediatric and 0.39 ± 0.59 in adult. However, there is no significant difference in mean change of BCVA between the two groups (*p* = 0.42). The mean BCVA before the surgery was 0.67 ± 0.23 in FU < 36 months group and 0.93 ± 0.64 in FU ≥ 36 months group, with a significant difference (*p* = 0.014). While, the BCVA after surgery was not significantly different between the two groups (*p* = 0.325;), also there is no significant difference in mean change of BCVA after surgery between the two groups (*p* = 0.28). The representative images for anterior segment images after surgery are presented in Fig. 3.

### Postoperative complications

The most significant postoperative complications in the early/intermediate period (from 1 day to 1 months after surgery) were transient intraocular haemorrhage in 2/77 eyes (2.6%) and early vitreous incarceration in 2 eyes (2.6%) with no statistical difference between adult and pediatric (Table 2). Both cases of transient intraocular haemorrhage were mild and developed by postoperative day 1, but were significantly or completely resolved within 1 week with the patient being semi-immobilised on a bed. Neither of these patients developed complications such as increased IOP or corneal blood staining. One case of early vitreous incarceration occurred on postoperative day 2 and the other occurred on postoperative day 5. Both patients underwent anterior vitrectomy, which successfully resolved the early vitreous incarceration.

**Table 2:** Early and late postoperative complications

Complications	NO. of eyes (%)
*Early postoperative complications*	
Transient intraocular hemorrhage	2 (2.6%)
Vitreous incarceration	2 (2.6%)
Total	4 (5.2%)
*Late postoperative complications*	
Retinal detachment	6 (7.8%)
IOL dislocation^a^	3 (3.9%)
Total	9 (11.7%)

^a^One eye with IOL dislocation required IOL replacement

*IOL* Intraocular lens

In the late postoperative period (> 1 months after surgery), retinal detachment occurred in 6/77 eyes (7.8%) within 12 to 53 months after IOL implantation, with a mean ± SD of 31 ± 15 months. In two eyes, retinal detachment was caused by postoperative ocular trauma. All of the eyes with retinal detachment underwent surgical treatment. IOL dislocation occurred in 3/77 eyes (3.9%) in the late postoperative period. Two of these involved mild dislocations, which did not need further treatment (Fig. 2B). The other eye experienced severe IOL dislocation with breakage of the polypropylene suture after experiencing ocular trauma at 50 months after surgery (Fig. 2C). The IOL was explanted and replaced with a new IOL. None of the 77 eyes experienced spontaneous breakage of the polypropylene suture during the follow-up period.

**Fig. 4:**
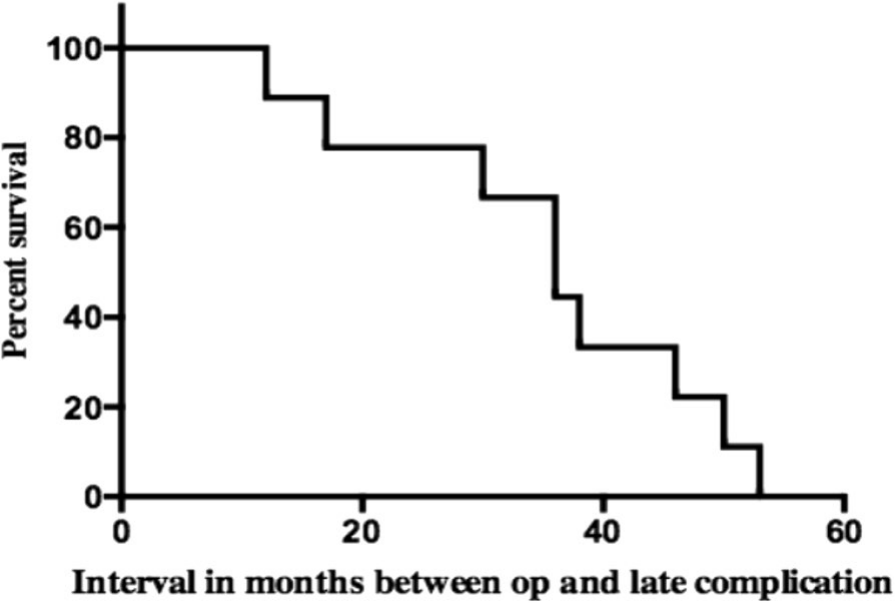
Cumulative timing of late postoperative complication following scleral sutured IOLs from time of surgery. The cumulative survival (vertical axis) shows as 100 the total number of cases that had late postoperative complication (9 eyes of 77 reported cases). The horizontal axis shows timing of events in months from time of surgery

The Fig. 4 shows the Kaplan–Meier curve for the time to occurrence of late complications. The Cox proportional hazards regression model showed that history of postoperative eye trauma was a significant risk factor for postoperative complications (*p* < 0.05), even after adjusting for age, gender, lens status, primary aetiology, and duration of follow-up.

When we take age into consideration, there is no statistical difference between adult and pediatric in occurrence of retinal detachment (*p* = 0.764) and IOL dislocation (*p* = 0.70). Follow-up times were also compared, no statistical difference between adult and pediatric in occurrence of retinal detachment (*p* = 0.061) and IOL dislocation (*p* = 0.194) in FU < 36 months group and FU ≥ 36 months group.

## Discussion

The optimal timing of lensectomy with primary SF-PC-IOL surgery is still not widely agreed. Many ophthalmologists believe that the minimum age for cataract extraction surgery is about 2 to 3 years in patients with congenital lens subluxation [10–12]. Literature reviews have suggested that SF-PC-IOLs should be implanted in patients after the age of 3 [13, 14]. However, because of the delicate situation of younger children and the technical complexity of surgery at a young age, many studies only performed SF-PC-IOLs in children aged 7 to 12 years [15, 16]. In our study, like the study by Asadi et al. [17], the earliest age of primary SF-PC-IOL implantation was 5 years old. In the present study, SF-PC-IOL implantation was performed in five 5-year-old children, and they all had a stable long-term prognosis.

In our follow-up, the mean ± SD BCVA improved significantly after surgery at the last visit (*P* < 0.001) in all case. Marginally significant difference of postoperative BCVA was found between pediatric group and adult group, which pediatric group was with a better BCVA. Therefore, we think receiving surgery in young age might improve visual prognosis. It might be due to the effect that lensectomy with primary SF-PC-IOL surgery can correct severe refractive error and high astigmatism, leading to an earlier intervention and better outcome of visual function training in children.

In the early postoperative period, transient intraocular haemorrhage occurred in 2/77 eyes (2.6%). Both of these cases may be related to scleral suturing of a PC-IOL, which requires the needle to be passed through vascular tissue. In both cases, the bleeding resolved spontaneously within 1 week of surgery. Other early complications were early vitreous incarceration, which occurred in 2 eyes (2.6%).

In the late postoperative period, retinal detachment occurred in 6 eyes (7.8%) within 12 to 53 months after IOL implantation. For lensectomy with a primary SF-PCIOL, retinal detachment is a potentially devastating postoperative complication. Retinal detachment seems to occur more frequently after SF-PC-IOL implantation than with other procedures. We think it might be due to the greater manipulation of the posterior segment or vitreous in this procedure than in other procedures. In a retrospective case-series, Gundula et al. [18] reported an incidence of 6.3% in their retrospective series of 63 eyes over 12 months. In the study by Johnston et al. [6], retinal detachment occurred in 10/24 patients (4%) with congenital lens dislocation. It was also reported that retinal detachment was more frequent in patients with Marfan syndrome than in patients with simple ectopia lentis [19]. However, in our study, the incidence of retinal detachment was not significantly different between Marfan syndrome and simple ectopia lentis.

Ophthalmologists have also focused on IOL dislocation caused by suture-related problems, such as suture knot exposure and suture degradation, after SF-PC-IOL implantation. The incidence of IOL dislocation due to suture breakage ranged from 17 to 28% in prior reports, and generally occurred 4 to 10 years after surgery [15]. Using electron microscopy, Parekh et al. [20] concluded that the surface properties of the positioning holes lead to cutting of the suture and subsequent subluxation of the PCIOL. Meanwhile, Prize et al. [21] observed five patients with PC-IOL suture breakage, and found that the extent of 10–0 polypropylene suture degradation differed among these five patients, and might represent another cause of IOL dislocation. However, in our series, there were no cases of spontaneous suture breakage causing IOL dislocation. Meanwhile, of three patients who experienced postoperative IOL dislocation, only one required IOL replacement. The ocular trauma in this patient was caused by domestic violence. Our findings are similar to those reported by Mimura et al. [22], who found no cases of spontaneous 10–0 polypropylene suture breakage among 16 patients followed up for > 10 years. Therefore, suture breakage may be related to the surgeon’s ability to place the suture. Nevertheless, it is important for patients to avoid ocular trauma after surgery.

Our study involved a long duration of follow-up and large sample sizes for patients undergoing primary SF-PC-IOL implantation to treat congenital lens subluxation. The long follow-up after surgery and the large sample size helped us assess the rate of complications and BCVA after surgery. Our findings suggest that implantation of a SF-PC-IOL provides good visual outcomes in patients with congenital lens subluxation and may improve the patient’s quality of life as a result of their improved visual function. However, there are several concerns regarding SF-PC-IOLs. In particular, retinal detachment and IOL dislocation affected some patients. Therefore, patients should be observed regularly to detect and treat possible complications for several years after surgery. This is particularly important for children with active lifestyles.

Congenital lens subluxation is most commonly found and treated in children. Therefore, surgery-induced tissue injury should be minimised and a favourable long-term prognosis is particularly important. Lensectomy combined with SF-PC-IOL implantation is a safe and effective treatment option for congenital lens subluxation.

## Data Availability

Original analyzed data of the follow-up clinical datasets (excluding patient information) are available from the corresponding author on reasonable request.
